# Draft Genome Sequence of *Salmonella enterica* subsp. *enterica* Serovar Typhimurium Q1

**DOI:** 10.1128/genomeA.01151-17

**Published:** 2017-10-19

**Authors:** Inga Eichhorn, Karsten Tedin, Marcus Fulde

**Affiliations:** Institute of Microbiology and Epizootics, Centre for Infection Medicine, Freie Universität Berlin, Berlin, Germany

## Abstract

Here, we report the draft genome sequence of *Salmonella enterica* subsp. *enterica* serovar Typhimurium strain Q1. The draft genome contains 4,793,493 bp in 149 contigs.

## GENOME ANNOUNCEMENT

The genus *Salmonella* comprises a heterogenous group of enteric pathogenic bacteria that are considered to belong to either a specialist or a generalist class. The specialists are strongly host adapted and confer a pathology that goes beyond self-limiting gastrointestinal infections, leading to abortive and/or systemic clinical pathologies. Typical representatives are the human-specific *S. enterica* subsp. *enterica* serovars Typhi (the causative agent of typhoid fever) and Paratyphi, as well as bovine-adapted *S. enterica* subsp. *enterica* serovar Dublin and pig-restricted *S. enterica* subsp. *enterica* serovars Choleraesuis and Parasuis. In contrast, nontyphoidal *S. enterica* subsp. *enterica* (NTS) serovars, such as *S*. Typhimurium and *S*. Enteritidis, are routinely isolated from a variety of different warm- and cold-blooded host species (1, 2). Infections with NTS serovars generally lead to local and self-limiting gastrointestinal infections in immunocompetent patients, as well as to typhoid-fever-like symptoms in immunocompromised individuals, neonates, and infants (1, 3–5). Thus, due to its zoonotic potential and exceptional role as a foodborne pathogen, NTS serovars are a paradigm for the (https://www.cdc.gov/onehealth/).

Despite the large number of *Salmonella* serovars, clinical pictures, and disease severity, the pathogenic mechanisms are very similar and depend in large part on virulence factors encoded within two large genomic islands, termed *Salmonella* pathogenicity islands 1 and 2 (6–8). It is widely accepted that horizontal gene transfer (HGT) is the main driver of *Salmonella* pathogenicity evolution, bacterial fitness, and host adaption and that lysogenic conversion of temperate phages has had a significant contribution. The most prominent examples of lysogenic phages in the genus *Salmonella* are the P2-like phages SopEΦ, Fels-1, and Fels-2 and the lambdoid prophages Gifsy-1, Gifsy-2, and Gifsy-3 (9–11).

Here, we provide the draft genome sequence of *S*. Typhimurium serovar Q1. Serologically, Q1 is a strain of *S*. Typhimurium, which was isolated from the feces of a human patient suffering from food poisoning and later cured of endogenous prophage (12). Q1 was believed to be phage and plasmid free (13), rendering it an excellent candidate strain for studies of HGT *in vitro* and *in vivo*. However, an uncharacterized, inducible bacteriophage infective for *S. enterica* subsp. *enterica* serovar Gallinarum was later observed (14). Using PHASTER, a Web-based online tool for identifying phage and phage-like sequences in bacterial genomes, we identified Gifsy-2 in the genome of *S*. Typhimurium Q1 (15, 16).

The 300 bp paired-end reads were generated using the Illumina MiSeq platform. The reads were *de novo* assembled into contigs with a minimum size of 200 bp using MIRA version 4.0 (17). A total of 149 contigs were generated ranging from 248 bp to 541,223 bp and resulted in a total genome size of 4,793,493 bp. The cumulative G+C content of the genome assembly was 52.2%. Gene annotation was performed using the RAST annotation server (18), which predicted 4,667 coding DNA sequences, 91 tRNAs, 42 rRNAs, and 1 transfer-messenger RNA in the draft genome. Strain Q1 was assigned to sequence type 19 using the multilocus sequence type service for total-genome-sequenced bacteria from the Center for Genomic Epidemiology (19). Using ResFinder (20) and PlasmidFinder (21), neither resistance genes nor plasmids were identified. Consistent with this observation, no phenotypic resistance against 11 different antibiotics was noted using the bioMérieux Vitek 2 system.

### Accession number(s).

This whole-genome shotgun project has been deposited at DDBJ/ENA/GenBank under the accession number NNSK00000000. The version described in this paper is the first version, NNSK01000000.
